# Professor Ajita Chakraborty MB, DPM, FRCP (Ed.) FRCPsych: Formerly Director of Postgraduate Medical and Research Institute, Professor and Head of the Department of Neurology and Psychiatry, Calcutta, India

**DOI:** 10.1192/pb.bp.115.051995

**Published:** 2016-04

**Authors:** Debasis Bhattacharya, Rahul Bhattacharya

**Figure:**
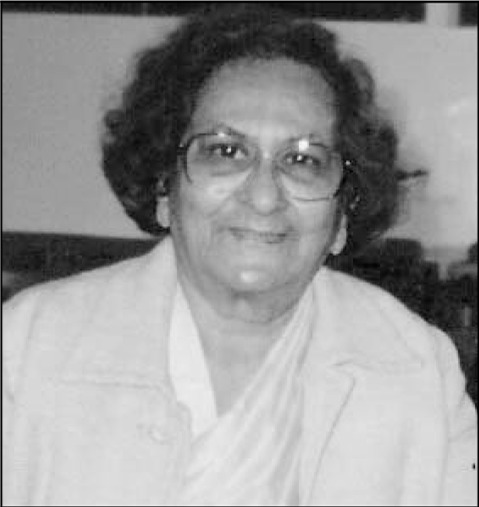


Ajita Chakraborty, who has recently died aged 88, pioneered the advancement of women in the field of psychiatry in India where she was the first woman to practise in the specialty. She was highly active in the Indian Psychiatric Society in which she served as general secretary (1967-1968), treasurer (1971-1974), vice president (1975) and finally, as a tribute to her indomitable leadership qualities and organisational skills, as president in 1976. Ajita had a keen interest in transcultural psychiatry. She first carried out pioneering epidemiological studies (with an impressive sample size of 13 335) around Calcutta exploring sociological and cultural perspectives. She noted that visual hallucinations (visions of gods and goddess) were common, particularly in women. This made making a differentiation between ‘pseudo’ and true hallucinations a challenge.^1^ She also studied the outbreak of Koro in eastern parts of India and proposed a hypothesis linking this disorder with displacement as well as loss of agricultural land and threats to cultural identity among the farming population.^2^ For several years she worked on evolving an indigenous school of psychotherapy that was well adapted for the people she treated most of her life. She was a member of the World Psychiatric Association, Transcultural Psychiatry Section, for 25 years. On her retirement from the editorial board of the journal *Transcultural Psychiatry*, the editor, Lawrence Kirmayer, thanked her for ‘unique and invaluable contributions’ to the journal over the years.

She was acutely aware of the disadvantages facing women in the professions in India, considering herself to be highly privileged at a time when, as she herself recalled ‘I was able to have advanced education and a professional career at a time when 90% of girls (in India) had no choice but marriage or family’. At the 5th World Congress of Psychiatry in Mexico City in 1971 she voiced her concerns that there were no sessions chaired by women and received widespread support. ‘All in all’, she wrote late in life ‘I think my life would have been easier if the gender was “right” ’. Although she did not wish to regard herself as a feminist, she acted as a role model for professional women in India at a time when feminism was still struggling to make its presence felt in the Western world.

Professor Ajita Chakraborty was born in Calcutta in the state of Bengal in 1926, at a time when the country was still a part of the British Empire. The city was the epicentre of British rule in India and had served as the capital of British India until 1911. Ajita's family had received Western education for generations and had been well connected, enterprising, urban middle-class with a streak of non-conformity that ran through generations. Throughout her early years Ajita was exposed to social inequalities, starting with the Bengal famine in 1943, followed by political unrest and riots in the backdrop of the Second World War. She lived through the granting of independence to India with the associated partition of the state of Bengal accompanied by unprecedented social displacement and uprooting that continued into the 1970s.

Ajita qualified from the Scottish Church College in Calcutta in 1944 (First Division) and enrolled for the Calcutta Medical College. Her entry group had an exceptionally high proportion of young women, with 10 girls and 50 boys. After qualifying as a doctor in 1950, she travelled to the UK for training in psychiatry, as the specialty was not yet established in India. After obtaining postgraduate qualifications in medicine and psychiatry, she returned to India in 1960 as the first female psychiatrist in the country. Early political struggles with the rising force of communism in Bengal and professional contact with psychoanalysis left her, as Ashis Nandy wrote in the foreword to her memoirs, ‘both anti-Freud and anti-communist’.^3^

Many of Professor Chakraborty's students consider themselves exceptionally fortunate to have had her as their teacher. She belonged to a rare breed of psychiatrists who critically evaluate psychiatric terminology and concepts, particularly exploring their cross-cultural validity, researching and collaborating internationally. She stimulated us through intellectual debate and introduced us to the idea of appraising psychiatric theories. She was instrumental in facilitating a rich interaction between psychiatrists, sociologists, psychotherapists and intellectuals, promoting independent thinking in her students. She continued to be a mentor to me (D.B.) throughout my career for over three decades. Many eminent Indian psychiatrists now working both in India and abroad, particularly in the UK, owe their professional expertise to their early training with her.

Anjita Chakraborty died on 8 May 2015.
